# Surgical treatment for antiplatelet intracerebral hemorrhage (SAP-ICH): protocol for a prospective cohort study of emergency surgery for severe spontaneous intracerebral hemorrhage patients on long-term oral antiplatelet treatment

**DOI:** 10.1186/s41016-020-00225-x

**Published:** 2021-01-11

**Authors:** Jun Wu, Xinguo Sun, Qingyuan Liu, Maogui Li, Shanwen Chen, Jiantao Wang, Youquan Wang, Peng Guo, Xiong Li, Lei Peng, Pengjun Jiang, Nuochuan Wang, Rui Guo, Shuzhe Yang, Yong Cao, Bo Ning, Cang Liu, Fuzheng Zhang, Jingping Li, Yanan Zhang, Shuo Wang

**Affiliations:** 1 Department of Neurosurgery, Beijing Tiantan Hospital, Capital Medical University, No.119 South 4th Ring West Road, Fengtai District, Beijing, 100070 China; 2 China National Clinical Research Center for Neurological Diseases, Beijing, People’s Republic of China; 3 Department of Neurosurgery, Binzhou People’s Hospital, Binzhou, 256610 Shandong China; 4 Department of Neurosurgery, Beijing Shunyi District Hospital, No. 3 Guangming Nan Street, Shunyi District, Beijing, 101300 China; 5 Department of Neurosurgery, Beijing Anzhen Hospital, Capital Medical University, No.2 Anzhen Road, Chaoyang District, Beijing, 100029 China; 6 Department of Neurosurgery, Beijing Pinggu District Hospital, 59 Xinping Bei Lu, Pinggu District, Beijing, China; 7 Department of Neurosurgery, Beijing Chaoyang Hospital, Capital Medical University, No.8 Gongti South Road, Chaoyang District, Beijing, 100020 China; 8 Department of Neurosurgery, Beijing Friendship Hospital, Capital Medical University, No.95 Yongan Road, Xicheng District, Beijing, 100020 China; 9 Department of Blood Transfusion, Beijing Tiantan Hospital, Capital Medical University, No.119 South 4th Ring West Road, Fengtai District, Beijing, 100070 China; 10 Department of Neurosurgery, Guangzhou Red Cross Hospital, Jinan University, Guangzhou, 510220 Guangdong China

**Keywords:** Severe spontaneous intracranial hemorrhage, Long-term antiplatelet treatment, Emergency surgery, Complications

## Abstract

**Background:**

Despite the capability of emergency surgery to reduce the mortality of severe spontaneous intracranial hemorrhage (SSICH) patients, the effect and safety of surgical treatment for severe spontaneous intracranial hemorrhage (SSICH) patients receiving long-term oral antiplatelet treatment (LOAPT) remains unclear. In consideration of this, the cohort study is aimed at figuring out the effect and safety of emergency surgery for SSICH patients on LOAPT.

**Methods:**

As a multicenter and prospective cohort study, it will be conducted across 7 representative clinical centers. Starting in September 2019, the observation is scheduled to be completed by December 2022, with a total of 450 SSICH patients recruited. The information on clinical, radiological, and laboratory practices will be recorded objectively. All of the patients will be monitored until death or 6 months after the occurrence of primary hemorrhage.

**Results:**

In this study, two comparative cohorts and an observational cohort will be set up. The primary outcome is the effect of emergency surgery, which is subject to assessment using the total mortality and comparison in the survival rate of SSICH patients on LOAPT between surgical treatment and conservative treatment. The second outcome is the safety of surgery, with the postoperative hemorrhagic complication which is compared between the operated SSICH patients on and not on LOAPT. Based on the observation of the characteristics and outcome of SSICH patients on LOAPT, the ischemic events after discontinuing LOAPT will be further addressed, and the coagulation function assessment system for operated SSICH patients on LOAPT will be established.

**Conclusions:**

In this study, we will investigate the effect and safety of emergency surgery for SSICH patients on LOAPT, which will provide an evidence for management in the future.

**Ethics and dissemination:**

The research protocol and informed consent in this study were approved by the Institutional Review Board of Beijing Tiantan Hospital (KY2019-096-02). The results of this study are expected to be disseminated in peer-reviewed journals in 2023.

**Trial registration:**

Name: Effect and safety of surgical intervention for severe spontaneous intracerebral hemorrhage patients on long-term oral antiplatelet treatment.

ChiCTR1900024406. Date of registration is July 10, 2019.

## Background

With high mortality and morbidity, spontaneous intracerebral hemorrhage is acute, severe hemorrhagic stroke [1]. For the patients suffering from severe spontaneous intracerebral hemorrhage (SSICH), emergency surgery may provide a means of saving their life [1–7]. Over the past few decades, the morbidity of such ischemia diseases as coronary heart disease has been on the increase with population aging, which may leave nearly a quarter of spontaneous intracerebral hemorrhage patients needing long-term oral antiplatelet treatment (LOAPT) [8]. However, the emergency surgery intended for patients on LOAPT carries a high level of risk to develop intraoperative and postoperative hemorrhagic complications [9, 10]. Up to now, the relative evidences were given only for selective operation, which recommended discontinuation of antiplatelet medications before surgery [1, 9, 11–15], which may hinder emergency surgery for SSICH patients on LOAPT; thus, there was no evidence about the emergency surgery for SSICH patients on LOAPT. Due to the rapid progression of SSICH and the potential risk of newly emerging and re-emerging ischemic events, however, it is usually impossible to discontinue antiplatelet treatment until coagulation function is restored. Once these complications occur, the patients would be faced with an even more catastrophic consequence [16]. Therefore, it is imperative for neurosurgeries and neurologists to develop an appropriate treatment strategy for SSICH patients on LOAPT.

Meanwhile, it is worth noting that there is still no effective strategy developed to assess the coagulation function of surgical patients on LOAPT. Given antiplatelet medication can have a significant impact on hemostasis and coagulation, the risk of intraoperative hemorrhage and postoperative intracranial bleeding (PIB) is a major focus because they can cause fatality [1, 2]. Though the preoperative screening of coagulation function could be effective in protecting patients from developing hemorrhagic complications, those commonly used methods are not applicable to assess the coagulation function of patients on LOAPT as the antiplatelet medication mainly affects platelet function, rather than clotting factors. Notably, there are various tools, such as thromboelastogram (TEG), reported to aid medical professionals in assessing the platelet function in the cardiac surgery and intracranial traumatic injury [17, 18]. Therefore, they have a potential to assist with the development of suitable treatment strategy for SSICH patients on LOAPT.

In addition to hemorrhagic complications, ischemic complications are also a problem worthy of consideration during the management of SSICH patients on LOAPT due to the hypercoagulable state caused by discontinuing antiplatelet medications. Besides, even though patients are stable after receiving appropriate treatment, it remains debatable to resume antiplatelet treatment. Therefore, there are many SSICH patients who have a history of antiplatelet treatment still stopping antiplatelet treatment after primary hemorrhage, which makes them under high risk of ischemic complications.

According to a previous study, the outcome after spontaneous intracerebral hemorrhage is poor, and the mortality could exceed 30% [1, 19]. Nevertheless, considering a lack of clarity on the effect and safety of emergency surgery for SSICH patients on LOAPT, there is no evidence gathered to support clinical decision-making. Therefore, a prospective cohort study will be conducted to investigate the safety and effect of emergency surgery for SSICH patients on LOAPT, based on which a scientific strategy will be proposed for the selection of an appropriate treatment.

## Methods

### Study design

**Fig. 1:**
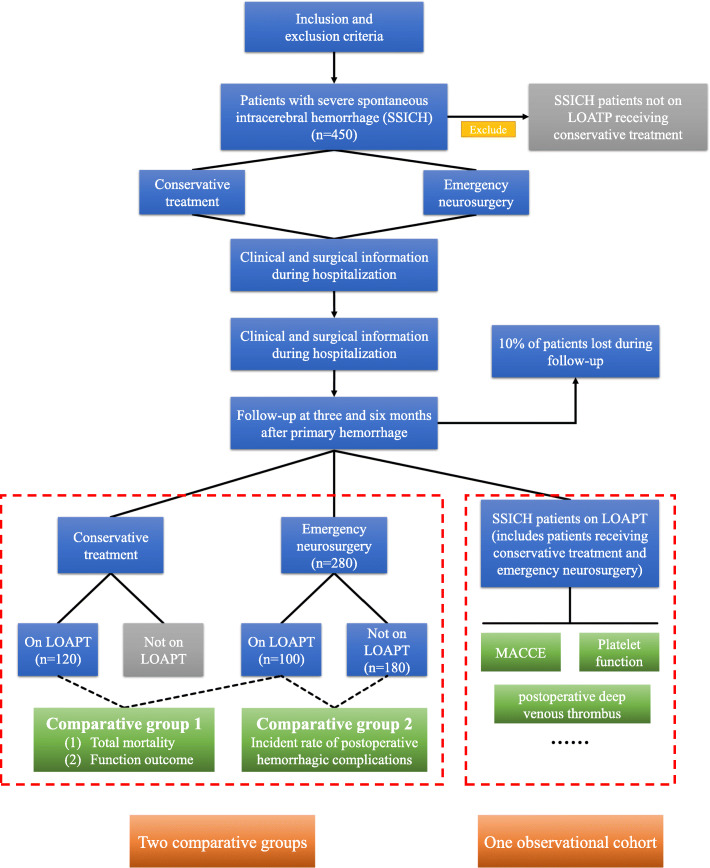
Research flow chart

Surgical treatment for antiplatelet intracerebral hemorrhage (SAP-ICH) is an ongoing and multicenter study; the flow chart of which is shown in Fig. 1. Beginning in September 2019, this trial is scheduled to be completed by December 2022. Due to unknown and hugely potential intracranial hemorrhage complications after neurosurgery, ethical issues need to be taken into account; therefore, the randomized controlled trial was not suitable currently. The purpose of this study is to evaluate the effect and safety of emergency surgery for SSICH patients on LOAPT, with the focus placed on four aspects: (1) the outcome of operated SSICH patients on LOAPT; (2) the risk of intraoperative and postoperative hemorrhagic complications; (3) the risk of developing postoperative ischemic diseases for SSICH patients on LOAPT; (4) the methods to assess the coagulation state of operated SSICH patients on LOAPT.

In order to achieve the research objective, it is necessary to recruit all SSICH patients except those not on LOAPT receiving conservative treatment. The inclusion and exclusion criteria are detailed in Tables 1 and 2, respectively. Three cohorts, including two comparative groups and one observational group (Fig. 1), will be established. Firstly, in order to measure the effect of emergency surgery, a comparison will be drawn of the outcomes and complications between SSICH patients on LOAPT receiving emergency surgery and those receiving conservative treatment. Secondly, in order to evaluate the safety of emergency surgery, hemorrhagic complications will be compared between operated SSICH patients who are on LOAPT and those not on LOAPT. Thirdly, the clinical characteristics and coagulation function of SSICH patients on LOAPT will be analyzed to assess the risk of developing ischemic diseases after discontinuing antiplatelet treatment, based on which an effective approach to the assessment of coagulation state will be established for surgery.

**Table 1:** Inclusion criteria

Inclusion criteria: (1) 18–75 years old; (2) non-traumatic intracerebral hemorrhage; (3) severe intracerebral hemorrhage, which was defined as patients with supratentorial bleeding volume > 30 ml, infratentorial bleeding volume > 10 ml, midline shift > 1 cm, or large intraventricular hematoma; (4) Glasgow coma score (GCS) < 13; (5) family members agree to provide an informed written consent.

**Table 2:** Exclusion criteria

Exclusion criteria: (1) patients had cerebrovascular diseases, e.g., intracranial aneurysm or vascular malformation, and intracranial tumors, which were associated with hemorrhage; (2) hemorrhagic transformation of cerebral infarction; (3) hemorrhage caused by venous thrombosis; (4) patients with severe coagulation disorder, e.g., hemophilia; (5) patients with coagulation dysfunction caused by malignant tumor, hypohepatia, renal dysfunction, thrombocytopenia, coagulation diseases, and so on; (6) patients receiving other anticoagulation medications (vitamin K antagonist and new oral anticoagulants); (7) patients not on LOAPT who receive conservative treatment; (8) the patients who died before or on arriving at the hospital and within a short period (6 h) after admission.	

### Qualification of participating center

To avoid participant bias while ensuring the sufficient number of cases included to reach our conclusion, all patients were recruited at the same time, and 7 neurosurgical centers were carefully selected considering the following criteria. Firstly, each center covers at least two communities or works as referral units for hemorrhagic stroke. Secondly, the center can carry out various treatment for SSICH, including all varieties of surgery and conservative treatment. Thirdly, the center is capable to admit more than 100 SSICH patients annually. According to an incomplete data, a total of 500 SSICH patients were evaluated across the 7 centers in 2019. Meanwhile, each center can fully represent the level of diagnosis and treatment of SSICH at a national level. Therefore, the centers involved in this study are considered representative.

### Outcomes and study endpoints

All patients will be closely monitored until death or 6 months after the occurrence primary hemorrhage. In this study, the following outcomes will be taken into consideration (Table 3). The primary outcome is the total mortality, including in-hospital and out-of-hospital mortality. The second outcome is the postoperative hemorrhagic complication, and the PIB is our main focus. The functional outcome of patients is another outcome interesting us and will be assessed using modified Rankin scale (mRS). The postoperative adverse cardiovascular/cerebral events (ACCE) include the region of original stroke enlarging, new ischemic stroke occurring outside the operative region, myocardial infarction, repeated revascularization, and deep venous thrombus. The screening, evaluation, and follow-up schedules are detailed in Table 4.

**Table 3:** Outcomes to be taken in consideration

(1) Total mortality; (2) postoperative hemorrhagic complication; (3) patients’ functional outcome; (4) postoperative adverse cardiovascular/cerebral events (ACCE).

**Table 4:**
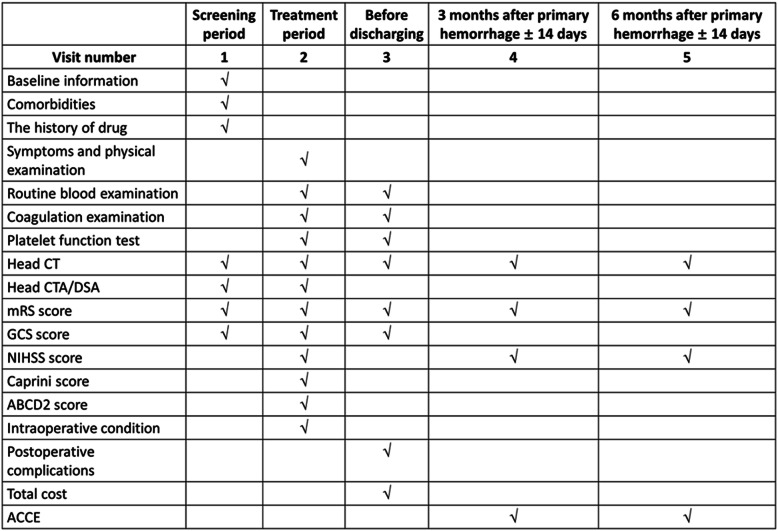
Screen, evaluation, and follow-up schedule

### Study definition

After admission, all of the patients would receive standardized care according to the guidelines. Severe spontaneous intracerebral hemorrhage (SSICH) is confirmed by both severe intracerebral hemorrhage radiologically and clinical coma (GCS < 13).

In definition, “on long-term oral antiplatelet therapy (on LOAPT)” refers to patients that received oral antiplatelet treatment on a continued basis for more than 7 days and discontinued antiplatelet medication no more than 7 days after the occurrence of hemorrhage. Antiplatelet therapy involved aspirin, clopidogrel, and dual antiplatelet treatment. Postoperative intracerebral bleeding (PIB) was defined as rehemorrhage within the operative region or new intracerebral hemorrhage associated with the operation on radiological examination.

### Thrombelastography (TEG) test and coagulation function assessment system

TEG is performed to monitor the function of platelet with the assistance of Thrombelastograph Coagulation Analyzer (TEG model 5000, Haemoscope Corporation, Niles IL, American). All parameters, including maximum amplitude (MA, mm), citric acid kaolin tracing (CK), reptilase tracing (A), aspirin tracing (AA), and clopidogrel tracing (ADP), are recorded through standard tracing. Based on MA, the rate of platelet inhibition was calculated using the formula as shown below:

$$ AA/ ADP\%=\frac{\mathrm{MA}\left(\mathrm{CK}\right)-\mathrm{MA}\left(\mathrm{A}\mathrm{A}/\mathrm{ADP}\right)}{\Big(\mathrm{MA}\left(\mathrm{CK}\right)-\mathrm{MA}\left(\mathrm{A}\right)} $$

where AA% represents the inhibition to platelet caused by aspirin, and ADP% indicates the inhibition to platelet caused by clopidogrel. Further with traditional coagulation test including bleeding time, prothrombin time, activated partial thromboplastin time, and fibrinogen as well as TEG, we plan to establish a combined coagulation function assessment system in order to assess the surgical risk facing SSICH patients on LOAPT, with the focus placed on PIB. Moreover, for the patients not on LOAPT, it is considered that these patients have normal platelet function and do not need to undergo further relative test.

### Data collection

In case the patients meet our criteria, all information, including the demography, clinical information, preoperative radiological data, intraoperative findings, and laboratory and TEG data, will be recorded on the case report form (CRF). After being discharged, all the patients were followed up on a regular basis by outpatient visit and on telephone 3 months and 6 months after primary hemorrhage, respectively. During each follow-up visit, the survival condition, mRS, ACCE, and medication will be recorded. If the patients feel unable to undergo radiological examination in the participating center, a follow-up cranial image will be required. Once radiological examination is completed, the patients’ imaging data will be transmitted to the Tiantan hospital (this center will work as an imaging interpretation center) to reconfirm several events, including SSICH, PIB, and postoperative ischemic disease. During the study, the researchers are required to protect privacy for the patients and ensure the confidentiality of their medical information. The information collection schedule is presented in Table 4.

### Data management

All data will be collected and stored using CRFs, and SHIP established a data management committee (DMC) in Tiantan hospital to monitor data quality and ensure data security. The clinical researcher associate (CRA) will make regular visit to each participating center to ensure the strict performance of research program. In case of any contradiction to the study protocol, the CRA will report this to the investigators promptly. Throughout this study, a summary meeting will be held every 3 months to discuss and solve any problem arising from the study or encountered by the patients.

## Results

China National Clinical Research Center for Neurological Diseases will be responsible for data analysis. Sample size was calculated by using PASS (Version 15, NCSS corporation, American). All sample size calculations were performed by conducting two-sided test, *α* = 0.05 and *β* = 80% (the degree of control = 80%). In this study, two comparison groups will be established, with the primary and secondary outcomes as the focus in this study. According to our unpublished data collected from 7 participating centers in 2019, for SSICH patients on LOAPT, the total mortality was about 30% for operated patients and 45% for the patients receiving conservative treatment, with the ratio of two groups reaching about 1:1.5. Moreover, for operated SSICH patients, the incidence of PIB was about 23% for patients on LOAPT and 10% for patient not on LOAPT, with the ratio of two groups reaching about 1:2.5. With regard to the primary outcome, our sample size was calculated as 196 (196 SSICH patients on LOAPT, operation group: *n* = 78; conservation group: *n* = 118). As for the secondary outcome, the sample size was 247 (247 operated SSICH patients, LOAPT group: *n* = 70; non-LOAPT group: *n* = 177). Moreover, in order to establish the coagulation function assessment system, the area under curve was expected to be 0.7, and the sample size was set to 92 (92 operated SSICH patients on LOAPT, PIB group: *n* = 23; non-PIB group: *n* = 69). In this case, a minimum of 387 SSICH patients (includes 118 patients on LOAPT receiving conservative treatment, 92 patients on LOAPT receiving emergency surgery, and 177 patients not on LOAPT receiving emergency surgery) may be required in this study to reach our research objective. Thus, to ensure final statistics, a total of 400 patients (patients on LOAPT receiving conservative treatment, *n* = 120; patients on LOAPT receiving emergency surgery, *n* = 100; patients not on LOAPT receiving emergency surgery, *n* = 180; Fig. 1) will be recruited. In this study, 10% of patients would be lost during follow-up. Finally, we decided to recruit 450 appropriate patients.

All statistical analyses were conducted with the assistance of SPSS (Version 22.0, IBM, American). The data was indicated as mean (x) ± standard deviation (SD) for normally distributed data, median, and interquartile range (IQR) for skewed distributed data or number (*n*) and percentage (%). In this study, two main comparative groups and an observational cohort will be established, as shown in Fig. 1. Firstly, in order to investigate the effect of emergency neurosurgery, a comparison will be performed between SSICH patients on LOAPT receiving conservative treatment and those receiving emergency neurosurgery. Secondly, in order to investigate the safety of emergency neurosurgery, a comparison will be performed between operated SSICH patients on LOAPT and those not on LOAPT. Thirdly, in order to investigate the intra/postoperative complications and the risk of ischemic disease after discontinuing antiplatelet treatment, the characteristics of SSICH patients on LOAPT will be analyzed; moreover, because the history of previous neural ischemic events or cardiac events could affect the risk of postoperative ischemic events, we would perform the subgroup analysis depending on this history to further investigate the effect of discontinuing antiplatelet treatment on the risk of ischemic disease. The difference will be compared by conducting χ^2^ test, Mann-Whitney *U* tests, or Student’s *t* tests. The independent risk factors were analyzed by logistic regression, while the factors with *p* < 0.10 in univariate analysis were entered into a multivariate logistic regression model. The result was indicated as odd ratio (OR) and 95% confident interval (CI). The receiver operating characteristic (ROC) curve analysis was performed to test the prediction value of different parameters and different methods. An area under the curve (AUC) in a ROC analysis of ≥ 0.7 is considered as a clinical utility, while *p* value of < 0.05 was treated as statistically significant.

### Ethics and public involvement

Patients or the public were not directly involved in design or conduct of the study. This study was granted approval from Institutional Review Board of Beijing Tiantan Hospital, and the research would be conducted in strict accordance with the declaration of Helsinki and Human Biomedical Research Ethical Issues. In this study, researchers are not able to use randomized controlled trials; thus, all recruited SSICH patients will not be affected by normal course of diagnosis and treatment. When SSICH patients are included, they will be followed up until 6 months after the occurrence of primary hemorrhage.

## Discussion

For the patients with SSICH, despite surgical intervention capable of saving life, the intraoperative and postoperative complications caused by an invasive procedure would lead to a secondary injury for those patients, which could result in a more catastrophic consequence, which explains why the previous evidences recommend against invasive procedures for the patients on antiplatelet medication. Over the most recent decades, however, due to the common use of antiplatelet medication, the rate of SSICH patients on LOAPT was on the increase annually, which prompts the exploration of appropriate treatment for SSICH patients on LOAPT. However, the problems we are facing now are neither appropriate treatment strategy nor assessment system of surgical risk for SSICH patients on LOAPT. At present, what confuses neurosurgeons is whether to perform emergency surgery on SSICH patients on LOAPT.

Considering that the mortality of all SSICH patients could reach as high as more than 30%, which is much higher among the patients with reduced platelet activity caused by LOAPT [19], it is essential to determine which treatment can benefit SSICH patient on LOAPT. SSICH tends to progress rapidly after primary hemorrhage, and the significant increase of intracranial pressure could put patients at high risk of developing cerebral hernia, which is a main cause of fatality [2, 20]. Though conservative treatments, known as mannitol, methylprednisolone, and hypertonic saline, may be effective in preventing patients from developing cerebral hernia, emergency surgery remains worth considering because this method can achieve quick and effective evacuation of hematoma, reduce high intracranial pressure, and prevent encephaledema-induced secondary cerebral hernia [4, 7, 21–23]. At present, the study about surgery for SSICH has yet to obtain any evidence suggesting that emergency neurosurgery can help reduce the mortality of SSICH patients on LOAPT. It is only in a study conducted by Stein et al. that the rate of in-hospital mortality was lower in the patients receiving surgery as compared to the patients receiving conservative treatment [24]. However, as this study is a retrospective study and most of the patients received only conservative treatment, the currently available data is still insufficient to support neurosurgeons in performing emergency surgery for SSICH patients on LOAPT, which explains why this study was conducted.

Another problem confusing neurosurgeon is how to assess the surgical risk of SSICH patients on LOAPT. The main surgical risk facing these patients stems from the coagulation disorder caused by antiplatelet medication [9, 11, 25]. It has been found out that there is a close association between coagulation condition and hemorrhagic complications. Notably, the common tests used in neurosurgical practice now are purposed simply to assess the coagulation function maintained by clotting factors. Nevertheless, these tests are insufficient to reveal the coagulation disorder caused by platelet function abnormality. Therefore, it is also imperative to establish a complete and applicable assessment system for SSICH patients on LOAPT. Though such tools as TEG and Verifynow were employed in previous study to assess platelet function, similar study was rarely conducted in neurosurgery. In a study conducted Holzmacher et al., the effect of platelet transfusion on prognosis in brain trauma patients was investigated, and the platelet function performed by TEG was examined [26]. Despite demonstrating that TEG can reflect the change of platelet function, this study failed in establishing the correlation between intra/postoperative complications and the result of TEG. Therefore, another crucial aim of this current study is to establish an effective and quick coagulation assessment system for SSICH patients on LOAPT, which is beneficial to neurosurgery.

The last focus of this study is ischemic complication after discontinuing antiplatelet medication. It is worth mentioning that the patients on antiplatelet medication are put at high risk of developing ischemic diseases including cardiovascular diseases and ischemic stroke. After a long period of discontinuing antiplatelet treatment, just for surgery, it is possible for these patients to develop ischemic diseases perioperatively or postoperatively [27]. This risk may be significantly higher for patients with ischemic stroke as neurosurgery could affect the cerebral blood flow and perfusion [2]. After the treatment of spontaneous intracranial hemorrhage, it is advisable to resume antiplatelet treatment. However, what confuses neurosurgeons is when and how to safely re-start antiplatelet medication. Moreover, there is currently still no relevant study reporting the incidence and risk factors of ACCE after neurosurgery in SSICH patients on LOAPT. Thus, credible evidence is needed to guide postoperative management. In this study, the patients will be followed up until 6 months after primary hemorrhage to investigate the risk of ACCE and the association between the discontinuation of antiplatelet treatment and ischemic complications. It is believed that this study will also provide an evidence to prevent SSICH patients on LOAPT from the development of ischemic complications and a basis for further investigation into the safe resumption of antiplatelet treatment in the future.

Based on our current study design, however, there remain some questions left unresolved. The first one is the indications of neurosurgery for SSICH patients on LOAPT. The second one is the effect of craniotomy, endoscopy, and minimally invasive surgery on SSICH patients on LOAPT. The third one is the advantage of different tools in assessing platelet function. The fourth one is when and how to resume antiplatelet treatment after neurosurgery. Surgical outcome and safety tend to be what neurosurgeons focus on. Despite the limitations to which our current study is subject, it is still believed that our study could provide a basis for the further exploration of appropriate treatment strategy for spontaneous intracranial hemorrhage patients on LOAPT.

## Conclusions

In this study, by analyzing the clinical features and outcome of SSICH patients on LOAPT, we will investigate the effect and safety of emergency surgery for these patients, which will provide an evidence for management in the future.

## Data Availability

Data sharing not applicable to this article as no datasets were generated or analyzed during the current study.

## Abbreviations

SAP-ICH: Surgical treatment for antiplatelet intracerebral hemorrhage
SSICH: Severe spontaneous intracranial hemorrhage
LOAPT: Long-term oral antiplatelet treatment
PIB: Postoperative intracranial bleeding
teg: Thromboelastogram
mRS: Modified Rankin scale
ACCE: Adverse cardiovascular/cerebral events
GCS: Glasgow coma score
MA: Maximum amplitude
CK: Citric acid kaolin tracing
A: Reptilase tracing
AA: Aspirin tracing
ADP: Clopidogrel tracing
CRF: Case report form
DMC: Data management committee
CRA: Clinical researcher associate
SD: Standard deviation
IQR: Interquartile range
ROC: Receiver operating characteristic
AUC: Area under the curve
